# Raman Spectroscopy for Pharmaceutical Quantitative Analysis by Low-Rank Estimation

**DOI:** 10.3389/fchem.2018.00400

**Published:** 2018-09-10

**Authors:** Xiangyun Ma, Xueqing Sun, Huijie Wang, Yang Wang, Da Chen, Qifeng Li

**Affiliations:** ^1^School of Precision Instrument and Opto-electronics Engineering, Tianjin University, Tianjin, China; ^2^State Key Laboratory of Precision Measurement Technology and Instruments, Tianjin University, Tianjin, China

**Keywords:** Raman spectroscopy, quantitative analysis, pharmaceuticals, low-rank estimation, chemometric model

## Abstract

Raman spectroscopy has been widely used for quantitative analysis in biomedical and pharmaceutical applications. However, the signal-to-noise ratio (SNR) of Raman spectra is always poor due to weak Raman scattering. The noise in Raman spectral dataset will limit the accuracy of quantitative analysis. Because of high correlations in the spectral signatures, Raman spectra have the low-rank property, which can be used as a constraint to improve Raman spectral SNR. In this paper, a simple and feasible Raman spectroscopic analysis method by Low-Rank Estimation (LRE) is proposed. The Frank-Wolfe (FW) algorithm is applied in the LRE method to seek the optimal solution. The proposed method is used for the quantitative analysis of pharmaceutical mixtures. The accuracy and robustness of Partial Least Squares (PLS) and Support Vector Machine (SVM) chemometric models can be improved by the LRE method.

## Intruduction

Raman spectroscopy is one of the vibrational spectroscopic techniques that has been commonly applied in quantitative analysis (Strachan et al., 2004; Numata and Tanaka, 2011; Ai et al., 2018). Being non-invasive and marker-free, it has been proved to be an effective tool in the field of physics, chemistry, and biology (Graf et al., 2007; Neugebauer et al., 2010; Ryu et al., 2012; Tan et al., 2017). Coupled with chemometrics methods, it has the advantages of high sensitivity and resolution in biomedical and pharmaceutical quantitative analysis.

The quantitative analysis based on Raman spectra at low signal-to-noise ratio (SNR) levels is still problematic (Li, 2008; Chen et al., 2014). Generally, a Raman spectrum can be divided into two parts: the signal containing desired information and the noise containing unwanted information. Basically, the latter may include photon-shot noise, sample-generated noise, instrument-generated noise, computationally generated noise, and externally generated noise (Pelletier, 2003). Due to the inherently weak property of Raman scattering, the noise will lead to a deterioration in SNR of Raman spectra, affecting the accuracy of quantitative analysis. For instance, data of online monitoring in limited integration time always tend to be inaccurate (Han et al., 2017; Virtanen et al., 2017).

Some approaches of preprocessing Raman spectra to minimize this problem have been proposed (Clupek et al., 2007; Ma et al., 2017), such as first and second derivatives (Johansson et al., 2010), polynomials fitting (Vickers et al., 2001), Fourier transform (Pelletier, 2003), and wavelet transform (Chen et al., 2011; Li et al., 2013). Among these approaches, wavelet transform can extract peak information and remove background noise, which has been the most widely used preprocessing method (Du et al., 2006). However, the processing of Raman spectra can be further optimized to improve the accuracy of pharmaceutical quantitative analysis.

In this paper, we introduce a simple and feasible Raman spectroscopic analysis method based on Low-Rank Estimation (LRE). Our experiments are implemented based on the Partial Least Squares (PLS) and Support Vector Machine (SVM) chemometric models. The aim of this experimental design is to enhance the quality of pharmaceutical quantitative analysis by significantly improving the accuracy and robustness of the chemometric models used.

## Materials and methods

Pharmaceutical substances (norfloxacin, penicillin potassium, and sulfamerazine) were purchased from Dalian Meilun Biotechnology Co., Ltd (China) and used without further purification. These substances were well blended in different proportions, pulverized, and compressed into three-component tablets. Other physical properties of these tablets (such as density, height, and diameter) were kept completely consistent. Mixed solutions were also prepared with methanol and ethanol in 100 different proportions. Raman spectral data were recorded by using a Renishaw inVia Raman spectrometer (Gloucestershire, U.K.). This system consisted of a 785-nm diode laser (~40 mW) and a 1,200 l/mm grating. In this work, the integration times of Raman spectra were 0.1–0.5 s.

PLS and SVM regression methods were used to model and predict pharmaceutical concentration of the samples based on their Raman spectra. Eighty-five samples were selected as the training set and the remaining 15 samples as the testing set, based on Kennard-Stone (KS) algorithm. The parameters of PLS and SVM models were tuned based on grid search algorithm. The optimal parameters were obtained by k-folder cross-validation.

The accuracy and robustness of above-mentioned chemometric models were further improved by conventional Wavelet Transform (WT) method and Low-Rank Estimation (LRE) method, respectively. In the WT method, the signals were split into different frequency components to remove simultaneously low-frequency background and high-frequency noise components. The Symlet wavelet filter (sym11, scale = 7) was optimally selected to provide the sharpest peaks associated with the analytes of interest. The LRE method was originally developed by our group in three-dimension to speed up Raman spectral imaging (Li et al., 2018). In this study, we used the LRE method in two-dimension to process the observed Raman spectral data matrix. In this method, the alternating least squares (ALS) algorithm is used to estimate the largest singular value of the matrix (Kroonenberg and Leeuw, 1980; Halko et al., 2011). The matrix estimation has two sets of parameters. Each set is estimated in turn by solving a least-squares problem and holding the other set fixed. After both sets have been estimated once, the procedure is repeated until convergence.

The Frank-Wolfe (FW) algorithm is applied in the LRE method to seek the optimal solution. Recently, the FW algorithm has been popularly used in machine learning due to its characteristics of simple implementation and modest memory requirement (Jaggi, 2013; Guo et al., 2017). The steps of the LRE method are detailed in Table 1.

**Table 1:** The detail steps of the LRE method.

**Algorithm:** The algorithm for the LRE method
**Input**: the raw Raman spectral data matrix *A*; the maximum number of iteration *N*, ranging from 5 to 20; the low-rank constraint factor *m*, ranging from 0.01 to 0.001;
1: **Initialize** *X^0^* = 0. *X^0^* is an initial solution of the algorithm.
2: **for** *i* = 0,1,…,*N* **do**, *a^*i*^* represents the *i*-th iteration of any variable *a*.
3: Compute the search direction *s*, *s*^*i*+1^ = ALS(*A*−*X*^*i*^)
4: Compute the step length *r*, $r^{i+1}=\arg\underset{r\in \left[0,1\right]}{\min}\left(A-\left(X^{i}+r\left(s^{i+1}-X^{i}\right)\right)\right)$
5: *X*^*i*+1^ = (1−*r*^*i*+1^)*X*^*i*^+*r*^*i*+1^*s*^*i*+1^
6: **stopping criterion:** $\frac{\text{ALS}\left(X^{i+1}\right)}{s^{i+1}}>m$
7: **end for**
8: The last iteration of *X* is the final solution of the LRE method.
**Output** *X*

Through being processed by the LRE method, the low-rank training and testing sets can be obtained from the raw training and testing data matrices, respectively. In general, an abundant data matrix can enhance the effect of the LRE method. When a number of testing spectral data is small, the training spectral data can be added to the raw testing data matrix as a supplement. The added spectral data are only used to strengthen the impact of the LRE method. The conventional regression models are applied to the low-rank training and testing sets to perform quantitative Raman analysis.

## Results and discussion

**Figure 1:**
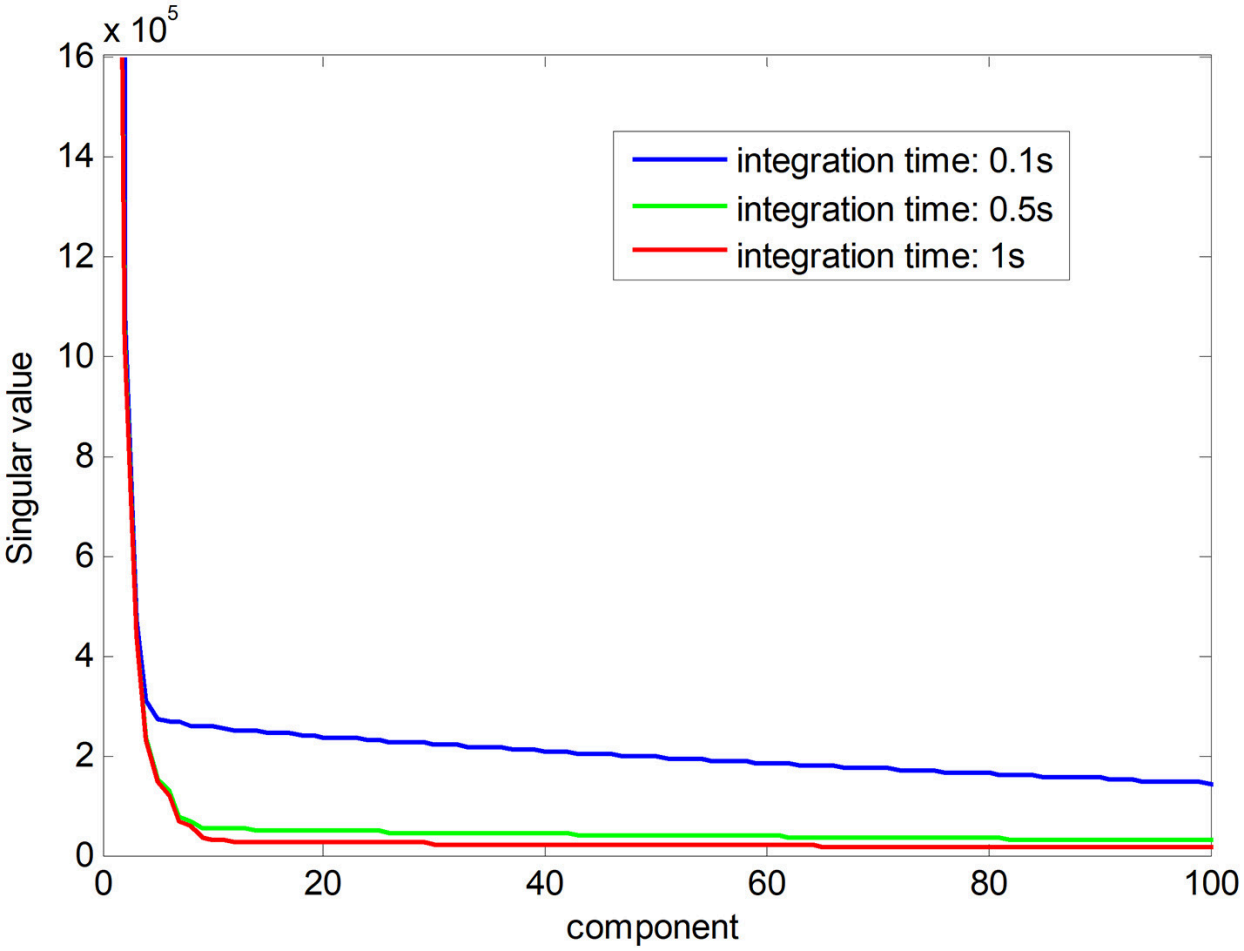
The ranks of the Raman spectra in different integration time.

Noise-free Raman spectral dataset is a low-rank matrix. In Figure 1, the red line shows the ranks of Raman spectral data matrix in an integration time of 1 s, suggesting that the Raman spectra have low-rank property when the noise is low. The low-rank property comes from high correlations among spectral signatures. Each spectral signature can be represented by a linear combination of a small number of pure spectral endmembers, which is known as linear spectral mixing model (Iordache et al., 2011; Golbabaee and Vandergheynst, 2012). The blue and green plots show singular values of the matrix in a shorter integration time, which implies that the ranks of Raman spectra increase with decreasing integration time owing to a greater proportion of the noise. The low-rank property can be used as a constraint to improve the accuracy of pharmaceutical quantitative analysis (Yi et al., 2017).

**Figure 2:**
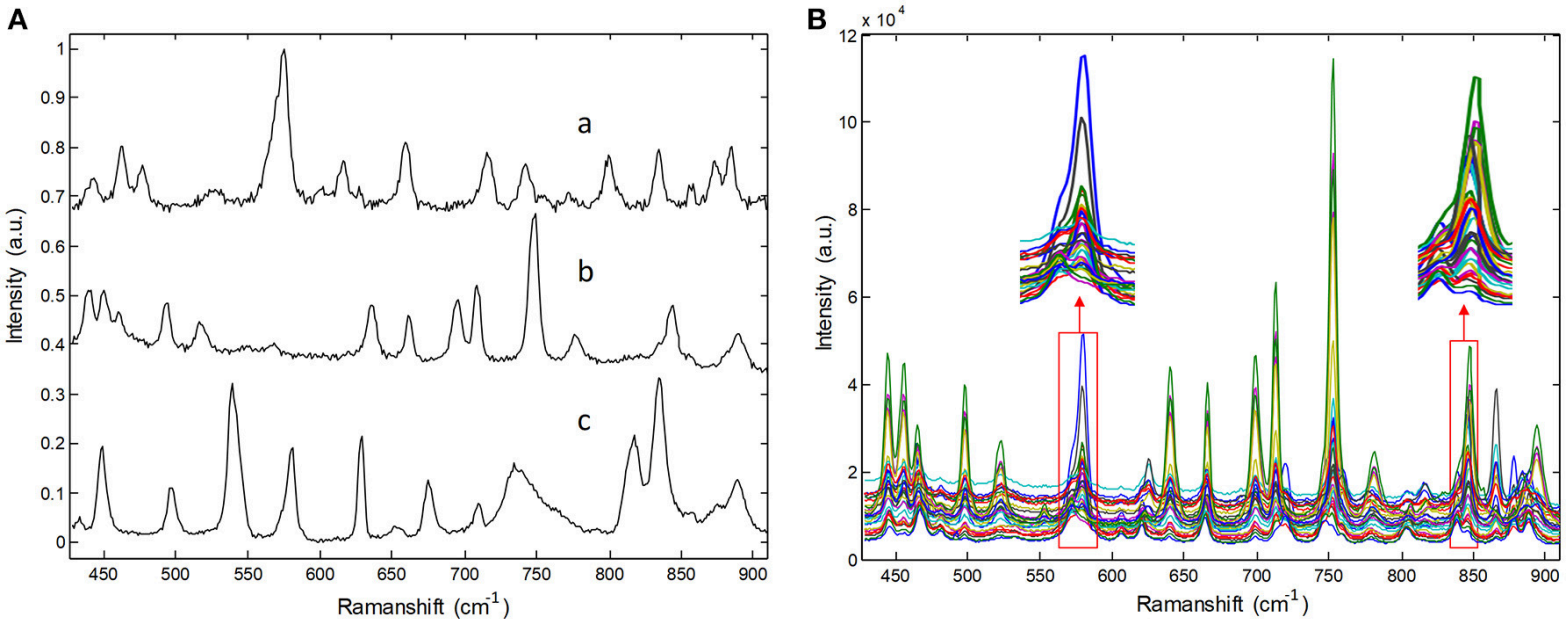
Raman spectra of **(A)** pure pharmaceuticals substances: (a) penicillin potassium, (b) norfloxacin, and (c) sulfamerazine, **(B)** their three component tablets containing different proportions in the integration time of 5s.

Raw Raman spectra recorded for three pure pharmaceutical substances are shown in Figure 2A. Thirty Raman spectra obtained from three-component tablets with different proportions are shown in Figure 2B. It is clear that each pharmaceutical component has its own special characteristic peaks. However, their respective Raman bands are overlapped. Particularly, Raman signals of lower-concentration component are almost swamped and covered by those of higher- concentration one, which represents a common problem in practice for biomedical and pharmaceutical quantitative analysis. For clarity, the Raman spectra in Figure 2B were collected in an integration time of 5 s, which have a high SNR. In our experiments, the integration times of Raman spectra are in the range of 0.1–0.5 s, which is over 10 times shorter than that shown in Figure 2. Under this condition, the spectral signals are weaker and have poor SNR.

**Figure 3:**
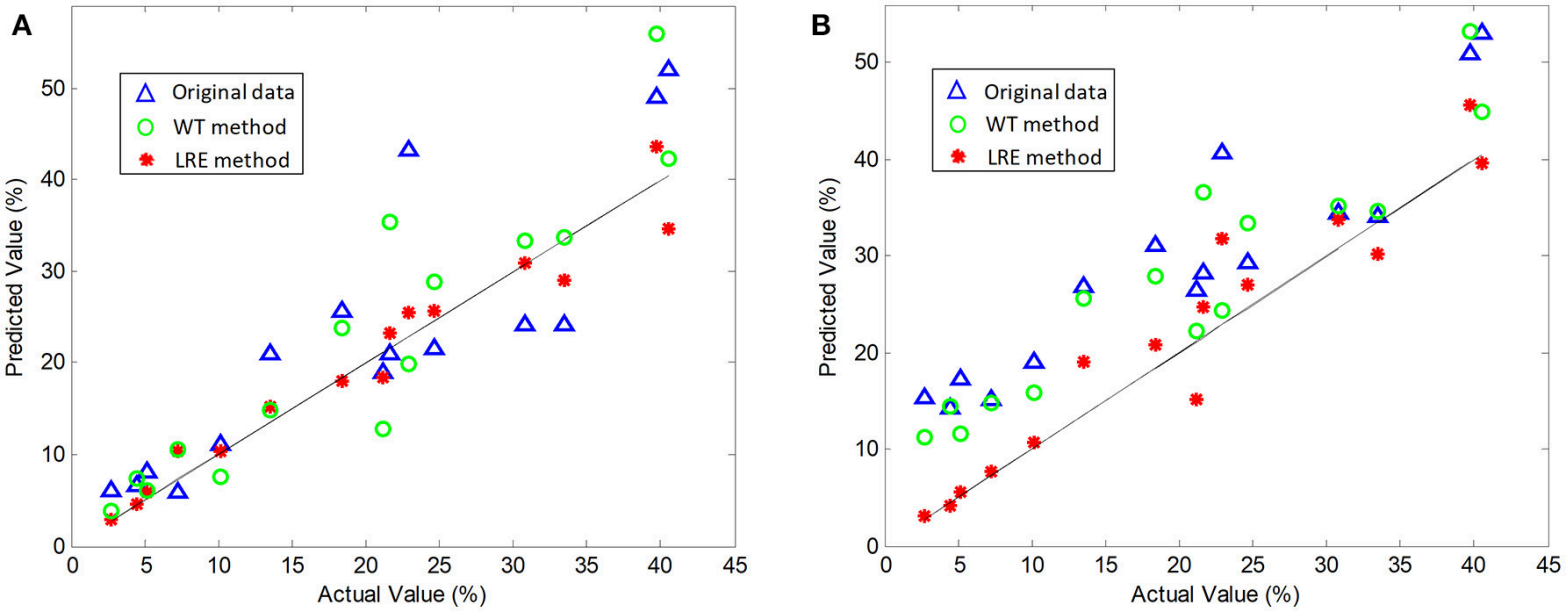
Actual vs. predicted values of norfloxacin based on the PLS **(A)** and SVM **(B)** model, where the black solid line are diagonals. Raw Raman spectra are collected in an integration time of 0.2 s.

The comparisons of predicted and actual values for norfloxacin are illustrated in Figure 3, which indicates the advantage of the LRE method for pharmaceutical quantitative analysis. The coefficient of determination (*R*^2^) and root mean square error (RMSE) of the chemometric models used for quantitative analysis of three pharmaceutical components are listed in Table 2. The unsatisfactory results of the raw spectral data show that the pre-treatment of Raman spectra is necessary. In this study, the LRE method and conventional wavelet transform (WT) method are applied to improve the accuracy of quantitative analysis. As shown in Figure 3, both the conventional WT and LRE methods can improve the predicted results. However, it is clear that the LRE method has a better performance than the conventional WT method in enhancing the prediction accuracy for pharmaceutical quantitative analysis.

**Table 2:** *R*^2^ and RMSE values of the chemometric models for three pharmaceutical components.

		**Norfloxacin**		**Penicillin potassium**		**Sulfamerazine**	
**Model**	**Methods**	***R*^2^**	**RMSE**	***R*^2^**	**RMSE**	***R*^2^**	**RMSE**
**PLS**	Raw	0.7504	0.0780	0.8692	0.1218	0.7323	0.0608
	WT	0.8598	0.0642	0.9548	0.0974	0.8862	0.0376
	LRE	0.9553	0.0259	0.9848	0.0522	0.9609	0.0225
**SVM**	Raw	0.8297	0.1097	0.8460	0.1264	0.8135	0.0679
	WT	0.8808	0.0841	0.9125	0.0821	0.8907	0.0444
	LRE	0.9558	0.0468	0.9749	0.0755	0.9701	0.0397

As shown in Table 2, the raw Raman spectra are all collected in an integration time of 0.2 s. The LRE method is significantly better than the conventional WT method in terms of *R*^2^ and RMSE for all components. Quantitation limit (QL) for each pharmaceutical substance is calculated. By definition in ICH guideline (ICH Harmonised Tripartite Guideline, 2005), QL is the lowest concentration of an analyte that can be quantitatively determined with suitable precision and accuracy. It is most often determined as 10 times the standard deviation of the noise from the blank. The LRE method can be used reliably with more than a 15-fold improvement of the practicalQL. Through being processed by the LRE method, QL values for norfloxacin, penicillin potassium, and sulfamerazine are 0.17, 0.13, and 0.19%, respectively. These results reveal that the LRE method can simultaneously improve the performance of quantitative analysis for pharmaceutical multi-component mixtures.

Table 3 lists *R*^2^ and RMSE values of the chemometric models used for quantitative analysis of norfloxacin in different integration times. The integration times of raw Raman spectra are 0.1, 0.2, and 0.5 s. Raman spectrum's SNR is always proportional to integration time. For evaluating spectral quality, the SNR is defined as the ratio of the peak value of the signal to the root mean square of the noise. For integration times of 0.1, 0.2, and 0.5 s, the average SNR of Raman spectra are 2.47, 3.66, and 6.21, respectively. *R*^2^ and RMSE values of the chemometric models for methanol in different integration times are listed Table 4. The average SNR of the Raman spectra in the integration times of 0.1, 0.2, and 0.5 s are 2.13, 3.34, and 5.89, respectively.

**Table 3:** *R*^2^ and RMSE values of the chemometric models for norfloxacin in different integration times.

		**0.1 s**		**0.2 s**		**0.5 s**	
**Model**	**Methods**	***R*^2^**	**RMSE**	***R*^2^**	**RMSE**	***R*^2^**	**RMSE**
	Raw	0.7286	0.0939	0.7606	0.0733	0.8731	0.0476
PLS	WT	0.8503	0.0630	0.8747	0.0627	0.9610	0.0446
	LRE	0.9496	0.0296	0.9626	0.0236	0.9784	0.0229
	Raw	0.7803	0.0959	0.8116	0.0894	0.9136	0.0781
SVM	WT	0.8673	0.0976	0.8987	0.0789	0.9251	0.0668
	LRE	0.9588	0.0449	0.9665	0.0229	0.9764	0.0210

**Table 4:** *R*^2^ and RMSE values of the chemometric models for methanol in different integration times.

		**0.1 s**		**0.2 s**		**0.5 s**	
**Model**	**Methods**	***R*^2^**	**RMSE**	***R*^2^**	**RMSE**	***R*^2^**	**RMSE**
	Raw	0.7078	1.9980	0.8086	1.4655	0.8458	1.3075
PLS	WT	0.8311	0.6551	0.8776	0.5750	0.9178	0.4553
	LRE	0.9017	0.5794	0.9301	0.4692	0.9401	0.4117
	Raw	0.7158	0.8631	0.8382	0.7669	0.8813	0.6148
SVM	WT	0.8361	0.7030	0.8701	0.6204	0.9428	0.4506
	LRE	0.9277	0.6417	0.9628	0.5112	0.9768	0.3964

As shown in Tables 3, 4, the accuracy of the quantitative analysis raises with increasing SNR. According to *R*^2^ and RMSE values, it can be proved that the LRE method has a better performance than the conventional WT method. The degree of improvement is higher for low-SNR Raman spectra, which indicates that the LRE method has good noise immunity.

In summary, all predicted results of the Raman spectra preprocessed by the LRE method are in good agreement with corresponding actual values. This method can be applied to improve the accuracy of quantitative analysis based on both PLS and SVM models. It is unrelated to the selection of chemometric models. The LRE method is not restricted by the state of a sample, meaning that it is applicable to both solid and liquid samples. Therefore, it can be regarded as an efficient tool with satisfactory prediction accuracy for pharmaceutical quantitative analysis, especially in the case of low-SNR spectra.

## Conclusion

The LRE method has been successfully applied in Raman spectroscopy for pharmaceutical quantitative analysis. It is a simply and feasibly method that can improve the accuracy and robustness of PLS and SVM chemometric models. Our data show that the LRE method has advantages in improving *R*^2^ and RMSE for quantitative analysis of pharmaceutical multi-component mixtures, especially in the case of low-SNR spectra. The LRE method will promote the development of Raman spectroscopy in biomedical and pharmaceutical quantitative analysis.

## Author contributions

XM participated in the lab work, supervising lab work, interpretation of data, drafting the manuscript, performing the statistical analysis. XS participated in the lab work, interpretation of data, drafting the manuscript, performing the statistical analysis. HW design of the work, interpretation of data. YW supervised the research, performing the statistical analysis. DC supervised the research, final approval of the version to be published. QL participated in the lab work, supervising lab work, final approval of the version to be published.

### Conflict of interest statement

The authors declare that the research was conducted in the absence of any commercial or financial relationships that could be construed as a potential conflict of interest.
